# Antitumor Activity and Induction of TP53-Dependent Apoptosis toward Ovarian Clear Cell Adenocarcinoma by the Dual PI3K/mTOR Inhibitor DS-7423

**DOI:** 10.1371/journal.pone.0087220

**Published:** 2014-02-04

**Authors:** Tomoko Kashiyama, Katsutoshi Oda, Yuji Ikeda, Yoshinobu Shiose, Yasuhide Hirota, Kanako Inaba, Chinami Makii, Reiko Kurikawa, Aki Miyasaka, Takahiro Koso, Tomohiko Fukuda, Michihiro Tanikawa, Keiko Shoji, Kenbun Sone, Takahide Arimoto, Osamu Wada-Hiraike, Kei Kawana, Shunsuke Nakagawa, Koichi Matsuda, Frank McCormick, Hiroyuki Aburatani, Tetsu Yano, Yutaka Osuga, Tomoyuki Fujii

**Affiliations:** 1 Department of Obstetrics and Gynecology, Faculty of Medicine, The University of Tokyo, Tokyo, Japan; 2 Oncology Research Laboratories, Daiichi Sankyo Co. Ltd., Tokyo, Japan; 3 Department of Obstetrics and Gynecology, Faculty of Medicine, Teikyo University, Tokyo, Japan; 4 Laboratory of Molecular Medicine, Human Genome Center, Institute of Medical Science, The University of Tokyo, Tokyo, Japan; 5 Helen Diller Family Comprehensive Cancer Center, University of California San Francisco, San Francisco, California, United States of America; 6 Genome Science Division, Research Center for Advanced Science and Technology, The University of Tokyo, Tokyo, Japan; 7 Department of Obstetrics and Gynecology, National Center for Global Health and Medicine, Tokyo, Japan; Taipei Medical University, Taiwan

## Abstract

DS-7423, a novel, small-molecule dual inhibitor of phosphatidylinositol-3-kinase (PI3K) and mammalian target of rapamycin (mTOR), is currently in phase I clinical trials for solid tumors. Although DS-7423 potently inhibits PI3Kα (IC_50_ = 15.6 nM) and mTOR (IC_50_ = 34.9 nM), it also inhibits other isoforms of class I PI3K (IC_50_ values: PI3Kβ = 1,143 nM; PI3Kγ = 249 nM; PI3Kδ = 262 nM). The PI3K/mTOR pathway is frequently activated in ovarian clear cell adenocarcinomas (OCCA) through various mutations that activate PI3K-AKT signaling. Here, we describe the anti-tumor effect of DS-7423 on a panel of nine OCCA cell lines. IC_50_ values for DS-7423 were <75 nM in all the lines, regardless of the mutational status of *PIK3CA*. In mouse xenograft models, DS-7423 suppressed the tumor growth of OCCA in a dose-dependent manner. Flow cytometry analysis revealed a decrease in S-phase cell populations in all the cell lines and an increase in sub-G1 cell populations following treatment with DS-7423 in six of the nine OCCA cell lines tested. DS-7423-mediated apoptosis was induced more effectively in the six cell lines without *TP53* mutations than in the three cell lines with *TP53* mutations. Concomitantly with the decreased phosphorylation level of MDM2 (mouse double minute 2 homolog), the level of phosphorylation of TP53 at Ser46 was increased by DS-7423 in the six cell lines with wild-type *TP53*, with induction of genes that mediate TP53-dependent apoptosis, including *p53AIP1* and *PUMA* at 39 nM or higher doses. Our data suggest that the dual PI3K/mTOR inhibitor DS-7423 may constitute a promising molecular targeted therapy for OCCA, and that its antitumor effect might be partly obtained by induction of TP53-dependent apoptosis in *TP53* wild-type OCCAs.

## Introduction

The phosphatidylinositol 3-kinase (PI3K)-AKT signaling pathway is frequently activated in various types of cancers, and several inhibitors that target this pathway have been developed as potential cancer therapeutics. The constitutive activation of the PI3K-AKT pathway results from various types of alterations, including the overexpression of receptor tyrosine kinases (RTKs), as well as mutations of *Ras*, the catalytic subunit p110α of phosphoinositide-3-kinase (*PIK3CA*), and *PTEN* [1]. Class I PI3Ks include four isoforms of the catalytic subunit (p110α, p110β, p110γ, and p110δ). Among these four isoforms, p110α is broadly mutated and predominantly activated in various types of human cancers, although p110β and p110δ might be selectively activated in certain tumors such as those with loss of PTEN function [2], [3]. Mammalian target of rapamycin (mTOR) is the catalytic subunit found in two distinct complexes: the raptor-containing complex mTORC1 and the rictor-containing complex mTORC2 [4]. AKT activates mTORC1 signaling and also phosphorylates other downstream proteins, including GSK3β, forkhead box-O transcription factors (FOXOs), and mouse double minute 2 homolog (MDM2) [5]. mTORC1 controls protein synthesis and cell proliferation via the phosphorylation of its downstream targets, 4E-BP1 and S6 kinase 1 (S6K1) [6]. Rapamycin and its analogs (rapalogs) block mTORC1 activity, but not mTORC2 activity [7]. One of the AKT downstream targets, MDM2, is a negative regulator of TP53 that induces its ubiquitination and subsequent degradation [8]. Although the cytostatic effects of PI3K pathway inhibitors have been reported in various types of cancers [9]–[12], targeting the PI3K pathway might induce cytotoxic effects by suppressing anti-apoptotic signals through the dephosphorylation of FOXOs and stabilization of TP53. It seems reasonable to suspect that targeting the PI3K-mTOR axis might be a promising therapeutic strategy to selectively induce apoptosis of cancer cells, especially those without mutations in *TP53*.

Epithelial ovarian cancer is a leading cause of death resulting from gynecological malignancies. Ovarian clear cell adenocarcinoma (OCCA) is the second most common cause of death from ovarian cancer, with a higher incidence in Asia, especially in Japan (>25%), than in other continents [13]. OCCA is derived primarily from ovarian endometriosis, and the clinical outcome is generally poor, owing to low response rates to conventional platinum-based chemotherapy [14]. Thus, novel therapeutic strategies are warranted to improve the clinical outcome of OCCA. In histological terms, ovarian serous adenocarcinoma (OSA) is the most common variant of ovarian carcinoma [15]. It is highly sensitive to platinum-based chemotherapy, with a primary clinical response rate of >70%. The mutational spectrum differs between OCCA and OSA, with *TP53* mutations observed in almost all (96%) OSA tumors, but in only 10% of OCCA tumors [15], [16]. In particular, mutations of *RB1* and *BRCA1/2* are much more common in OSA than in OCCA. However, *PIK3CA* mutations are more frequent in OCCA (>40%) than in OSA (<10%) [17]. Although mutations of *KRAS* and *PTEN* are rare (<10%), the overexpression of several RTKs has been reported in OCCA, including human epidermal growth factor receptor 2 (HER2) with a frequency of approximately 40% and cMET with a frequency of approximately 30% [18]–[21]. Taken together, these observations suggest that the RTK-PI3K/mTOR signaling axis might be broadly activated in OCCA.

DS-7423 is a novel, small-molecule compound that inhibits both PI3K and mTOR (mTORC1/2). It inhibits all class I PI3K isoforms with greater potency against p110α than against the other p110 isoforms. Relevant activity (IC_50_ <200 nM) was not observed in any of the 227 kinases tested, except for Mixed Lineage Kinase 1 (MLK1) and Never-In-Mitosis Gene A (NIMA)-related kinase 2 (NEK2). The compound is currently in phase I clinical trials for solid tumors. In this study, we evaluated its anti-tumor efficacy in a panel of OCCA cell lines. We focused in particular on the ability of DS-7423 to induce apoptosis, and on whether the apoptosis might be mediated by TP53.

## Materials and Methods

### Small-molecule compounds

The small molecule compound DS-7423 was provided by the Daiichi-Sankyo Company, Ltd (Tokyo, Japan). The drug information about DS-7423 is available on the ClinicalTrials.gov website (NCT01364844). The mTOR inhibitor rapamycin was purchased from Cayman Chemical (Michigan, USA).

### Cell lines

The OVTOKO, OVISE, OVMANA, RMG-I, OVSAHO, OVKATE, and OV1063 lines were purchased from the Japanese Collection of Research Bioresources (JCRB) Cell Bank (Osaka, Japan). The JHOC-7, JHOC-9, HTOA, JHOS-2, JHOS-3, and JHOS-4 cell lines were purchased from the RIKEN Cell Bank (Ibaraki, Japan). The TOV-21, ES-2, and SKOV3 cell lines were from the American Type Culture Collection (Manassas, VA). OVISE, OVTOKO, TOV-21G and ES2 were cultured in RPMI1640 medium containing 10% fetal bovine serum (FBS). OVMANA was cultured in RPMI medium containing 20% FBS, JHOC-7 in DMEM/F12 medium containing 10% FBS, JHOC-9 and RMG-I in DMEM/F12 medium containing 20% FBS, and SKOV3 in DMEM containing 10% FBS. The OVSAHO, OVKATE, OV1063, HTOA, JHOS-2, JHOS-3, and JHOS-4 lines were cultured in DMEM medium containing 10% FBS. The histological subtype of the SKOV3 cells was not unambiguously defined even after extensive analysis, although it was confidently identified as clear cell adenocarcinoma [22]. The immortalized epithelial cell line from an ovarian endometrial cyst was a generous gift from Dr. Satoru Kyo [23].

### Polymerase chain reaction (PCR) and sequencing

The mutational status of all nine OCCA cell lines was analyzed by PCR and direct sequencing. The PCR conditions and primers for the analysis of *PTEN* (exons 1–9) and *K-Ras* (exon 1 and 2) sequences were described previously [24]–[26]. The entire coding region of *PIK3CA* was analyzed by reverse transcription (RT)-PCR with LA-Taq according to the manufacturer's protocol (Takara BIO, Madison, WI) [27]. The PCR primers for analysis of *TP53* (exons 4–8) were described previously [28].

### Proliferation assays

Assays of the suppression of cell proliferation were performed with the Cell Counting Kit-8 using the tetrazolium salt WST-8 [2-(2-methoxy-4-nitrophenyl)-3-(4-nitrophenyl)-5-(2,4-disulfophenyl)-2H-tetrazolium, monosodium salt] (Dojindo, Tokyo, Japan) for the methyl thiazolyl tetrazolium (MTT) assay. Using 96-well plates, 2,000 cells were seeded on the appropriate medium and treated with increasing doses (0–2,500 nM) of DS-7423 or rapamycin for 72 h, starting from 24 h after seeding. Proliferation was quantified by monitoring the changes in the absorbance at 450 nm, which were normalized relative to the absorbance of cell cultures treated with DMSO alone.

### Immunoblotting

Cells were treated with DS-7423 or rapamycin for the indicated time and at the indicated concentration, and were then lysed in the cell lysis buffer (Cell Signaling Technology, Beverly, MA). Antibodies to total Akt, phosphorylation of Akt (p-Akt) (Ser473, Thr308), p-GSK3beta (Ser9), total S6, p-S6 (Ser235/236, Ser 240/244), p-4EBP1 (Thr37/46), p-FOXO1 (Thr24), p-FOXO3a (Thr32), p-MDM2 (Ser 166), p-TP53 (Ser15), cleaved-PARP, and PARP (Cell Signaling Technology, Beverly, MA), beta-actin (Sigma-Aldrich, St. Louis, MO), TP53 (Santa Cruz, CA, USA) and p-TP53 (Ser46) (Calbiochem, Billerica, MA) were used for immunoblotting, as recommended by the manufacturers. Signals were detected using BioRAD western blotting systems (BioRAD, Hercules, CA) with the detection reagents ECL advance and ECL select (GE Healthcare, Piscataway, NJ).

### Cell cycle analysis

Cells (5×10^5^) were seeded in 60-mm dishes and treated with DS-7423 for 48 h. Floating and adherent cells were collected by trypsinization and washed twice with phosphate buffer saline (PBS). Cells were resuspended in cold 70% ethanol and maintained at 4°C overnight. After being washed twice with PBS, cells were incubated in RNase A (0.25 mg/mL) (Sigma) for 30 min at 37°C, followed by staining with propidium iodide (PI; 50 μg/mL) (Sigma) at 4°C for 30 min in the dark. Cells were then analyzed using flow cytometry (BD FACS Calibur HG, Franklin Lakes, NJ). Cell cycle distribution was analyzed using CELL Quest pro ver. 3.1. (Beckman Coulter Epics XL, Brea, CA). All experiments were repeated three times.

### Detection of apoptosis by staining with annexin-V FITC

Cells (5×10^5^) were cultured in 60-mm plates for 24 h before treatment with either DMSO (control), and 156 nM DS-7423, or 2,500 nM DS-7423 for 48 h. Cells were trypsinized, washed twice with PBS, and then analyzed after double staining with annexin-V fluorescein isothiocyanate (FITC) (Abcam, Cambridge, MA) and PI. The apoptotic cell population was analyzed using flow cytometry. All experiments were performed three times.

### Ethics statement for animal experiments

This study was approved by Animal Care and Use Committee, Daiichi-Sankyo Pharmaceutical Co. Ltd. Athymic mice were maintained in an SPF (Specific Pathogen Free) facility according to our institutional guidelines, and experiments were conducted under an approved animal protocol.

### Tumor xenografts in nude mice

Specific pathogen-free female nude mice (BALB/cAJcl-nu/nu), 6 weeks old, were purchased from CLEA Japan, Inc (Tokyo, Japan). Subcutaneous xenograft tumors in the mice were established by the injection of a 100-μL suspension containing 5×10^6^ cells of the TOV-21, RMG-I, or ES-2 lines in PBS. Tumors were removed after exponential growth, cut into 3-mm pieces, and transplanted subcutaneously into other mice for RMG-I cells. DS-7423 was suspended in 0.5 w/v% Methyl Cellulose 400 solution (Wako Pure Chemical Industries, Ltd.) Oral daily administration of DS-7423 started 8–22 days later, following the injection of the cells (5–6×10^6^ cells/0.1 mL). One week after tumor transplantation, mice were assigned randomly to one of the three treatment regimens: (1) non-treated control, (2) DS-7423 (1.5 mg/kg), (3) DS-7423 (3 mg/kg), and (4) DS-7423 (6 mg/kg). Each treatment group consisted of five mice. DS-7423 was injected orally (p.o.) once a day. Tumor volumes (in mm^3^) were calculated by the formula: ([major axis] × [minor axis]^2^/2). After the treatment, the tumors were removed and analyzed by western blotting. Tumor weight (wet weight) was measured, and the average weight was calculated for each group.

### Semi-quantitative RT-PCR analysis

OCCA cells were treated with either DMSO or the indicated concentration of either DS-7423 or rapamycin for 24 h. Total RNAs of these cells were extracted with the RNeasy Mini Kit according to the manufacturer's instructions (QIAGEN, Valencia, CA). cDNAs were synthesized from total RNAs by using the Super Script III First-strand Synthesis SuperMix (Invitrogen, Carlsbad, CA). The exponential phase of the RT-PCR occurred between 15–30 cycles, and these cycles were monitored to allow semi-quantitative comparisons among the cDNAs developed from identical reactions. The primers and conditions for the amplification of *p53AIP1*, *p21,* and *GAPDH* sequences were described previously [29]. The PCR primers for *PUMA* were 5′-TGAGACAAGAGGAGCAGCAG-3′ (forward) and 5′-ACCTAATTGGGCTCCAT CTC-3′ (reverse). The primers for p53R2, TIGAR, GLS2, GADD45, 14-3-3 sigma and PAI-1 were described previously [30]–[35]. Each PCR regimen involved a 2-min initial denaturation step (94°C), which was followed by 15–30 cycles at 94°C for 30 s, then at 55°C for 30 s, and finally, at 72°C for 30 s using a Thermal Cycler Gene Atlas instrument (ASTEC, Fukuoka, Japan).

### Gene silencing

Cells were plated at approximately 30% confluence in 100-mm plates and incubated for 24 h before transfection with small interfering RNA (siRNA) duplexes at the concentrations indicated, using Lipofectamine 2000 RNAiMAX (Invitrogen, Carlsbad, CA) and Opti-MEM medium (Life Technologies, Grand Island, NY). The siRNAs specific for TP53 were purchased from Invitrogen. A negative control kit was used as a control (Invitrogen, Carlsbad, CA).

### Luciferase assay

Transfection was performed using Effectene reagent (QIAGEN, Valencia, CA) according to the manufacturer's recommendation. The TP53 expression plasmid (0.1 µg/µL) was cotransfected with pp53 TA Luc (0.25 µg/mL). The phRL CMV-Renilla plasmid (Promega, Madison, WI) was also transfected in all experiments as the internal control to normalize the transfection efficiency. The assays, each involving triplicate wells, were repeated three times.

### Statistical analysis

The data were expressed as means ± standard deviations of three independent determinations. The significance of the difference between two samples was analyzed using the Student's t-test, and a p-value of <0.05 was considered to denote a statistically significant difference.

## Results

### Genetic alterations and activation of the PI3K-AKT signaling pathway in OCCA cell lines

**Figure 1:**
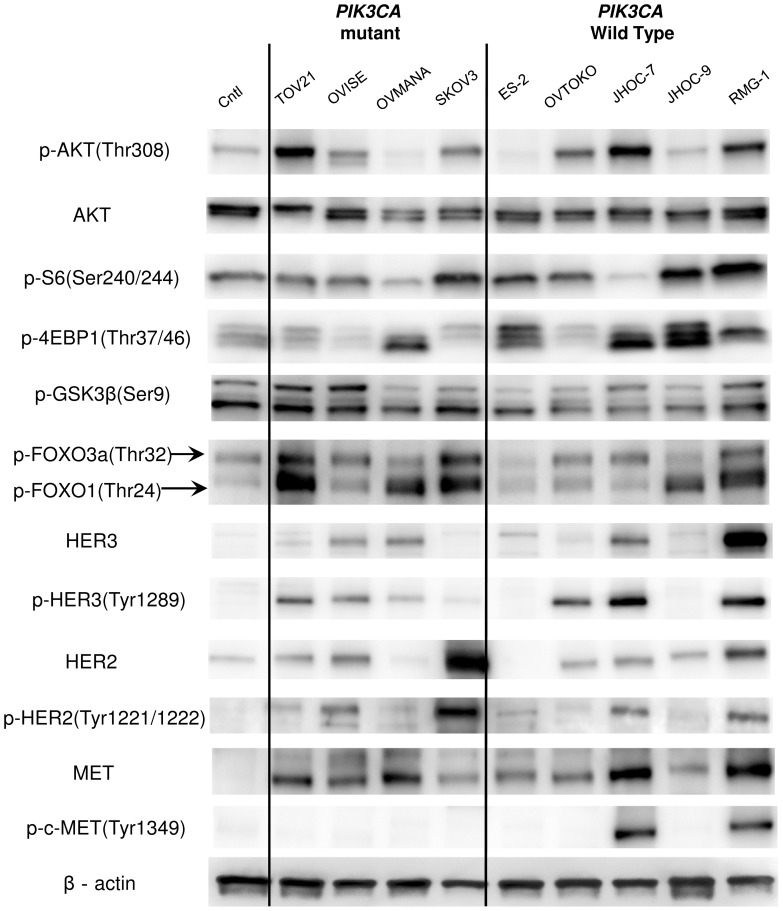
Phosphorylation and mutational status of genes that encode components of the RTK/Ras/PI3K pathway. Nine ovarian clear cell adenocarcinoma (OCCA) and a control (Cntl) cell line (immortalized epithelial cells from ovarian endometrioma) were lysed in cell lysis buffer and analyzed by western blotting. In general, most of the OCCA cell lines displayed higher levels of phosphorylation of Akt (Thr308) and its downstream targets (GSK3β, FOXO 1/3a, 4EBP1 and S6) than the respective levels of phosphorylation in the control line. The abundances and levels of phosphorylation of c-MET (Tyr1234/1235), HER2 (Tyr1221/1222), and HER3 (Tyr1289) were also evaluated. The mutational status of *PIK3CA*, *PTEN*, and *K-Ras* is shown for each cell line.

We evaluated the phosphorylation (p-) levels of the proteins in the PI3K-AKT pathway by using an immortalized epithelial cell line from an ovarian endometrial cyst as a control. AKT was phosphorylated at Thr308 in seven of the nine OCCA cell lines tested (Figure 1). The cell lines OVMANA and ES-2 had low levels of p-AKT (Thr308) (Figure 1). The phosphorylation levels of S6, 4E-BP1 and/or FOXO1/3a, the downstream targets of AKT, were upregulated in the OCCA cells, including OVMANA and ES-2.

Four of the nine cell lines possessed *PIK3CA* mutations (44%) (Figure 1 and Table S1), and one of these four, TOV-21G, also possessed mutations in *PTEN* and *K-Ras* (11%). *TP53* mutations were detected in three cell lines (33%) (Table S1). The mutational status of *PIK3CA* was not associated with the phosphorylation of AKT or proteins that act downstream of AKT. Next, the expression and phosphorylation levels of three RTKs (HER2, HER3, and MET), which have been reported to be overexpressed in OCCA, were evaluated. The levels of phosphorylation of both HER2 (Tyr1221/1222) and HER3 (Tyr1289) were correlated with the abundances of these two proteins (Figure 1). p-HER2 and p-HER3 levels were elevated in four (44%: OVISE, SKOV3, JHOC7 and RMG-I) and six (67%: TOV-21G, OVISE, OVMANA, OVTOKO, JHOC-7 and RMG-I) cell lines, respectively (Figure 1). The expression of MET was higher in all nine OCCA cell lines than in the control, although the level of p-MET was increased in only two cell lines (22%: JHOC-7 and RMG-I). Taken together, all the OCCA cell lines, except for ES-2 and JHOC-9, possessed one or more activating alterations in the RTK-PI3K genes examined (Figure 1 and Table S1). Each of the four cell lines with *PIK3CA* mutations showed concomitant activation of RTKs, defined as high levels of phosphorylation of HER2 and/or HER3.

### Anti-proliferative effect of DS-7423 in OCCA cell lines

**Figure 2:**
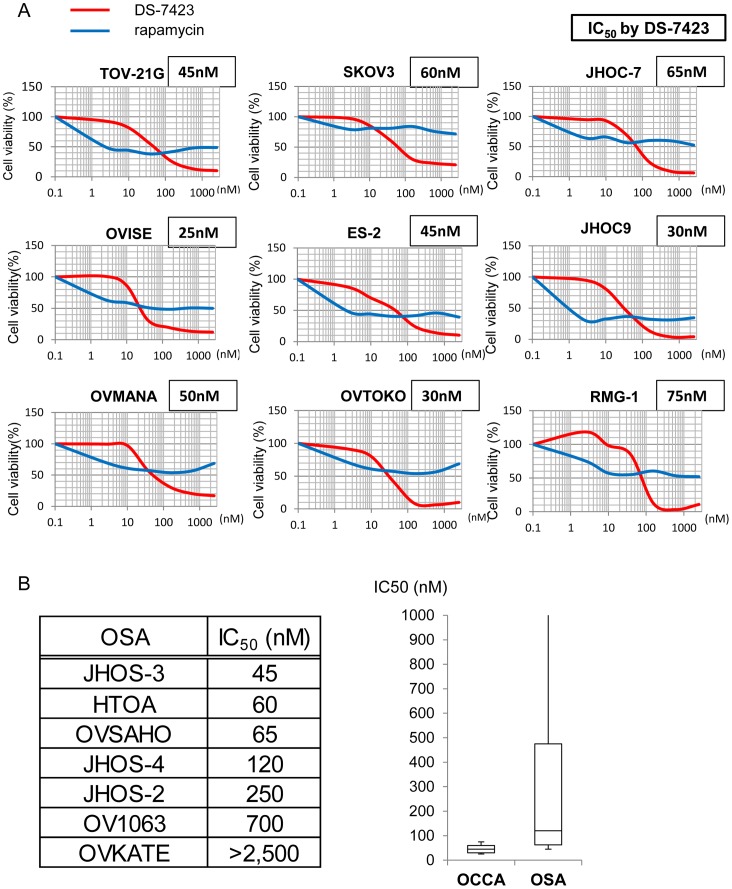
Inhibition of cell proliferation by DS-7423 and rapamycin. (A) Cell viability for each cell line was analyzed using the methyl thiazolyl tetrazolium (MTT) assay 72 h after treatment with DS-7423 or rapamycin at the doses indicated. The data were normalized relative to the value of the control cells. In all nine cell lines, DS-7423 suppressed cell proliferation more robustly than rapamycin when both were used at higher doses. (B) IC_50_ values for DS-7423 in seven ovarian serous adenocarcinoma (OSA) cell lines (left) were compared with those of nine OCCA cells (right). Four of seven OSA cells had IC_50_ values >100 nM, which is higher than that of any OCCA cells.

We tested the anti-proliferative effects of the dual PI3K/mTOR inhibitor, DS-7423, and the mTOR (mTORC1) inhibitor, rapamycin, in each of the nine OCCA cell lines. Exposure to 156 nM DS-7423 inhibited cell growth by 70%–97%, and the IC_50_ values for cell proliferation were 20–75 nM (Figure 2A). Dose-dependent growth suppression was more clearly induced by DS-7423 than by rapamycin in each of the nine cell lines (Figure 2A). The IC_50_ value was not reached with rapamycin at any of the concentrations tested (2.45–2,560 nM) in five (OVMANA, SKOV3, OVTOKO, JHOC-7 and RMG-I) of the nine OCCA cell lines. We also examined the effect of DS-7423 in seven OSA lines. The IC_50_ values with DS-7423 were >100 nM in four of these seven OSAs (Figure 2B). The ratio of resistant cell lines (IC_50_ >100 nM) was significantly higher in OSA cell lines (57%) than in OCCA cell lines (0%) (p = 0.019 by Fisher's exact test).

**Figure 3:**
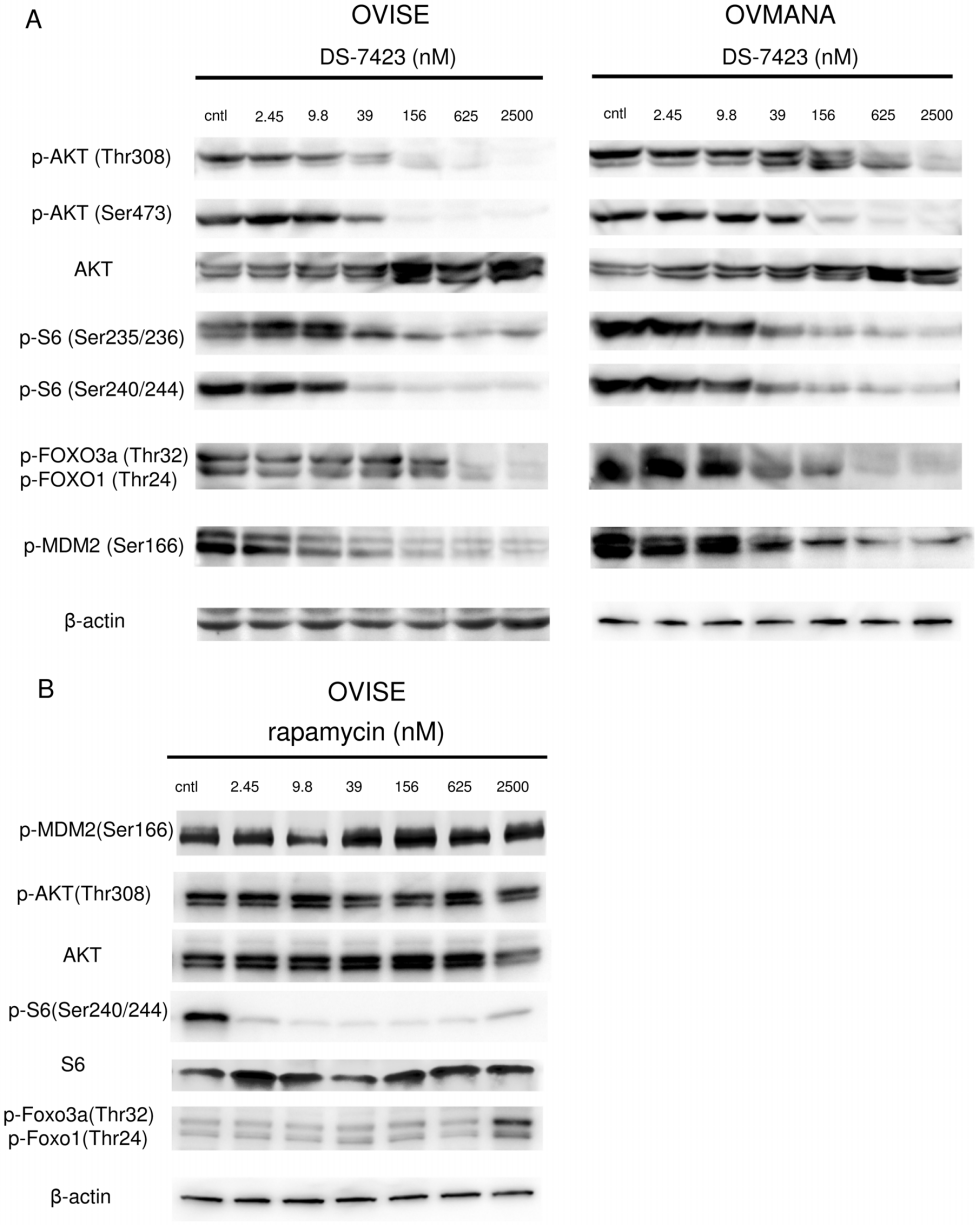
Inhibition of PI3K/mTOR signaling by DS-7423 and rapamycin in ovarian clear cell adenocarcinoma cell lines. (A) Immunoblotting of total protein extracts from OCCA cells (OVISE and OVMANA) treated with DS-7423 at concentrations ranging from 0 to 2,500 nM. (B) Immunoblotting of total protein extracts from OVISE cells treated with rapamycin at concentrations ranging from 0 to 2,500 nM.

We performed immunoblotting on the lysates prepared from the cells treated with DS-7423 or rapamycin. DS-7423 suppressed the phosphorylation of AKT (Thr308 and Ser473) and S6 (Ser235/236 and Ser240/244) at doses of 39–156 nM and higher (Figure 3A and Figure S1). DS-7423 suppressed the phosphorylation levels of the targeted proteins at comparable doses in the AKT pathway (AKT, FOXO1/3a, and MDM2) and mTORC1 pathway (S6). Rapamycin did not suppress p-Akt at any dose, and suppressed p-S6 at 2.45 nM or higher doses (Figure 3B). On the contrary, rapamycin increased the levels of p-FOXO3a and p-FOXO1 at 2,500 nM (Figure 3B).

**Figure 4:**
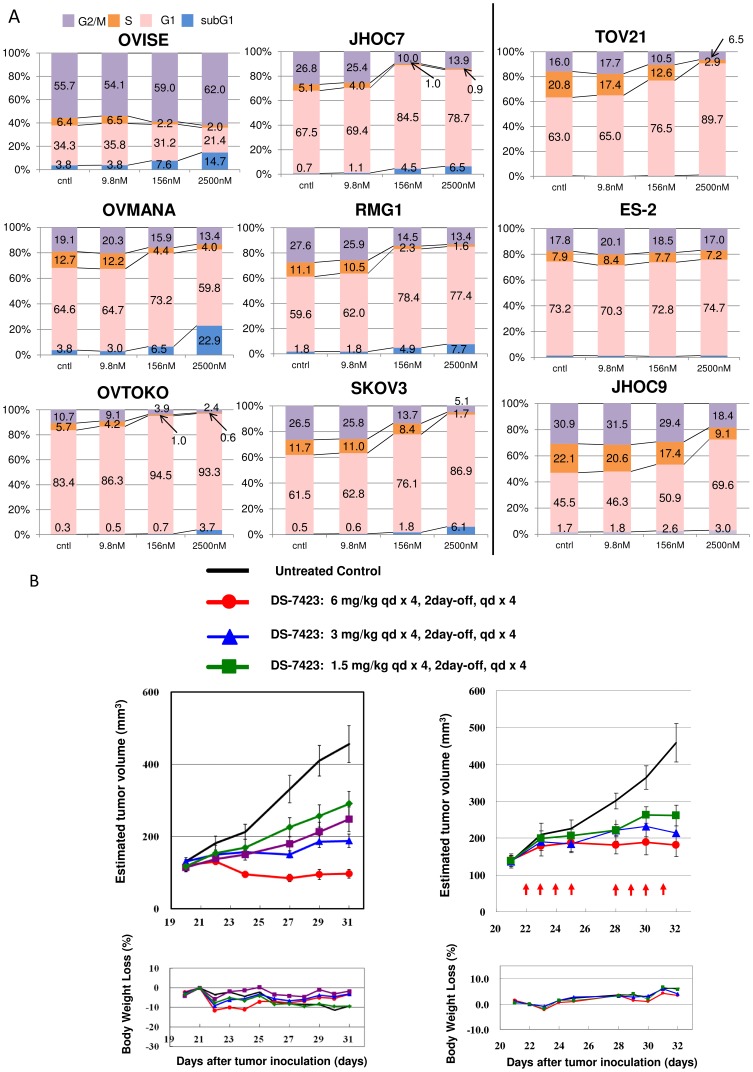
Flow cytometric analysis of the cell cycle in cancer cells treated with DS-7423, and *in vivo* demonstration of the anti-tumor effect of DS-7423 in nude mice. (A) Cells (5×10^5^) were seeded in the presence of 10% serum and treated with DS-7423 for 48 h at doses of 9.8 nM, 256 nM, or 2,500 nM. DS-7423 blocked OCCA cell cycle progression into the S phase in a dose-dependent manner. The relative size of the sub-G1 population was increased in six of the cell lines (left) but was not affected in the remaining three cell lines (right). (B) Subcutaneous xenograft tumors in athymic BALB/c mice were established following the injection of OCCA cells of either the TOV-21G (left) or RMG-I (right) cell lines. Mice were treated daily (5–7 days per week) at the indicated doses of DS-7423 (1.5, 3, or 6 mg/kg, 8–10 days). Each treatment group contained five mice. Estimated tumor volumes (upper graphs) and body weight losses (BWL) (lower graphs) were shown in the two OCCA cells. Tumor volumes were calculated by the formula {(major axis)*(minor axis)^2^/2} mm^3^. Groups were compared at the end of treatment. Points, mean; bars, standard deviation (SD); *p<0.05.

We conducted fluorescence-activated cell sorting (FACS)-based cell cycle analyses in OCCA cells treated with DS-7423. DS-7423 decreased the size of the S-phase population in the OCCA cells, although the change was weak in ES-2 cells. (Figure 4A). G1 arrest was predominantly observed in six of the nine cell lines. The sizes of sub-G1 populations increased in a dose-dependent manner in six of the nine cell lines, especially in OVISE and OVMANA cells.

### *In vivo* antitumor effect of DS-7423 in a mouse xenograft model

*In vivo* antitumor activity of DS-7423 in mice implanted with either TOV-21G cells or RMG-1 tumor pieces was examined. Oral daily administration of DS-7423 significantly suppressed the tumor growth of the xenografts of TOV-21G and RMG-I in a dose-dependent manner (Figure 4B). No significant adverse effects, including body weight loss of more than 10%, were observed in the mice examined (Figure 4B). Treatment with DS-7423 suppressed the levels of p-AKT (Thr308) and p-S6 (Ser240/244) in the TOV-21G and RMG-I xenografts (Figure S2A). Compared with TOV-21G and RMG-I xenografts, the anti-tumor effect of DS-7423 was weaker in xenografts with ES-2, for which the basal level of p-Akt (Thr-308) was low (Figure S2B).

### Induction of apoptosis by DS-7423 in TP53 wild-type cell lines

**Figure 5:**
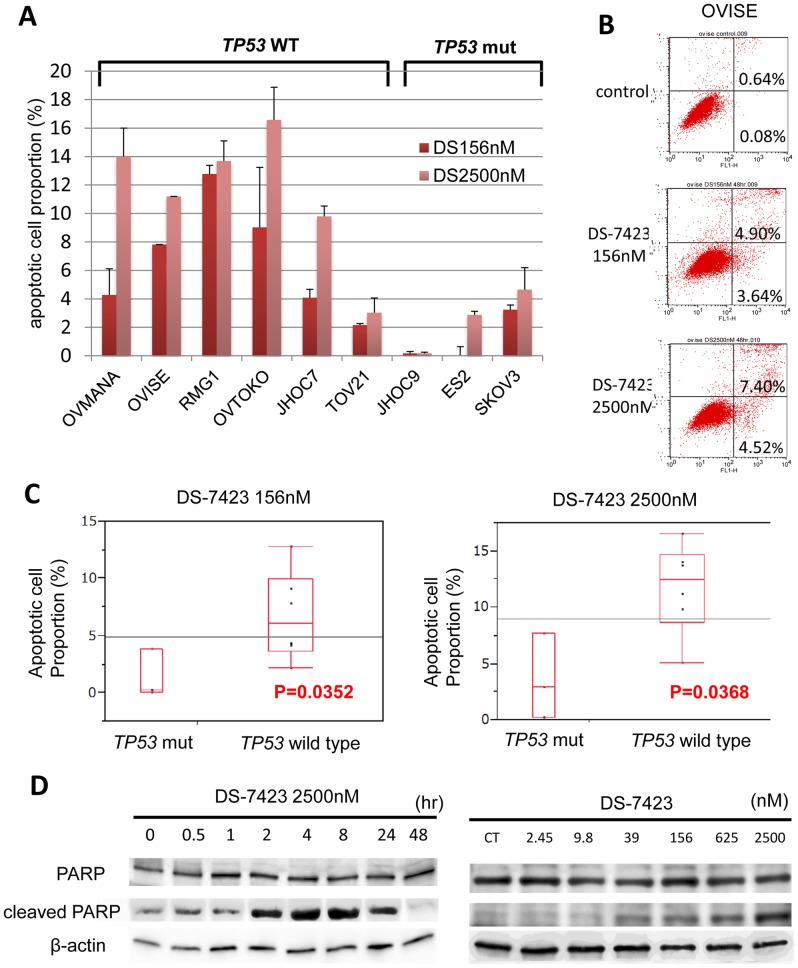
DS-7423–mediated induction of apoptosis in ovarian clear cell adenocarcinoma cell lines. (A) All nine OCCA cells were treated with DS-7423 at 156 or 2,560 nM for 48 h, and apoptotic cell proportion was evaluated using annexin-V fluorescein isothiocyanate (FITC) and propidium iodide (PI) double staining, followed by analysis using flow cytometry. The experiments were repeated 3 times, and each value is shown as the mean of 3 experiments ± standard deviation (SD). (B) The apoptotic cells were calculated using flow cytometry by counting the cell population in the right boxes. The example shown (OVISE cells) is representative of the results obtained for all the cell lines tested. (C) The proportion of cells rendered apoptotic by exposure to DS-7423 at 156 nM and 2,560 nM was significantly higher in OCCA cells without mutations in TP53 than in OCCA cells that carry mutations in TP53. (D) Cleaved poly(ADP-ribose) polymerase (PARP) induction was evaluated by immunoblotting in OVISE cells. OVISE cells were treated with DS-7423 at 156 nM for the times indicated (left) or for 4 h at the doses indicated (right).

The data collected from FACS analysis suggested that DS-7423 has a cytotoxic and cytostatic effect in certain OCCA cell lines. We combined the DS-7423 treatment (156 nM or 2,500 nM) with double staining with annexin-V FITC and PI to evaluate the proportion of cells that underwent apoptosis. DS-7423 at 156 nM induced apoptosis at 4–12% in five of the six cell lines that lacked mutations in *TP53* (Figure 5A and 5B). In these five cell lines, 2,500 nM DS-7423 induced apoptosis in 10–16% of the cells. In three cell lines with *TP53* mutations, DS-7423 did not induce apoptosis in >5% of the cells at any of the doses tested (Figure 5A). The size of the population of apoptotic cells was significantly higher in cells that lacked mutations in *TP3* when compared with cells with mutated *TP3* at either 156 nM (p = 0.0352) or 2,500 nM (p = 0.0368) DS-7423 according to the Student *t*-test (Figure 5C). Rapamycin did not induce apoptotic cell death in >5% of the OCCA cells, even at 2,500 nM. The percentage of apoptotic cells was significantly higher in OVISE cells treated with DS-7423 than that in those treated with rapamycin (Figure S3). This result indicates that mTORC1 inhibition alone is insufficient to induce apoptosis in OCCA cell lines. Immunoblotting analysis revealed that DS-7423 induced the cleavage of PARP within 2 h in OVMANA cells without mutations in *TP53* (Figure 5D). The induction of cleaved-PARP was observed at 39 nM, and the effect increased in a dose-dependent manner up to a concentration of 2,500 nM (Figure 5D).

### Induction of p-TP53 at Ser46 and expression of *p53AIP1* by DS-7423

**Figure 6:**
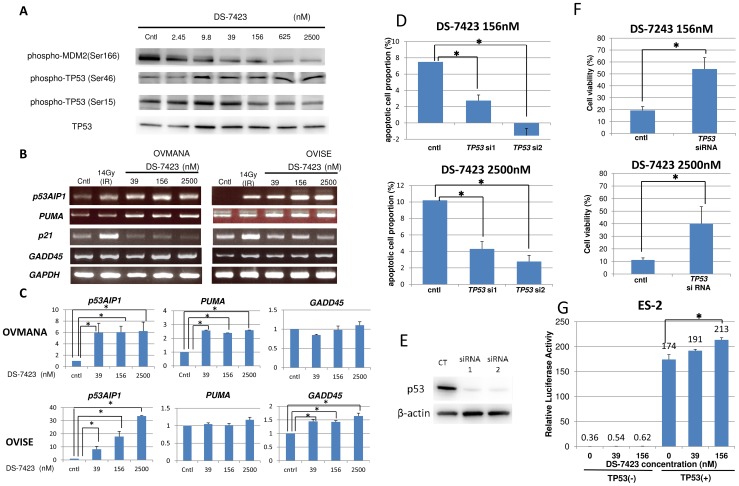
Induction of the phosphorylation of TP53 at Ser46 and the accumulation of transcripts of the genes targeted by TP53, which participate in TP53-mediated apoptosis. (A) Immunoblotting in OVMANA cells treated with DS-7423 at the indicated doses. Phosphorylation levels of MDM2 were inversely associated with p-TP53 at Ser46, but not with p-TP53 at Ser15. (B) Semi-quantitative RT-PCR in OVMANA and OVISE cells treated with DS-7423 at the indicated doses. Both *p53AIP1* and *PUMA* were induced by DS-7423. CT, untreated (negative) control; IR, irradiation at 14 Gy (positive control). GADD45 was induced in OVISE, but not in OVMANA cells. (C) Quantification of the semi-quantitative RT-PCR in (B). Each experiment was repeated 3 times, and each value is shown as the mean of 3 experiments ± SD. *p<0.05 (D) Effect of *TP53* knockdown on apoptosis induction by DS-7423. TP53 was knocked down by two independent siRNAs specific to *TP53* (siRNA1 and 2) in OVISE cells, which do not carry any mutation in *TP53*. The apoptotic cell population was evaluated using annexin-V staining, as described in Figure 5. The experiments were repeated 3 times, and each value is shown as the mean of 3 experiments ± SD. *p<0.05 (E) Suppression of TP53 expression by siRNAs was confirmed by immunoblotting. (F) Effect of *TP53* knockdown on cell proliferation by DS-7423 in MTT assay of OVISE cells. TP53 was knocked down by a siRNA1 specific to *TP53* and MTT assay was subsequently performed as in Figure 2. Knockdown of TP53 diminished the anti-proliferative effect caused by DS-7423 on OVISE cells. The experiments were repeated 3 times, and each value is shown as the mean of 3 experiments ± SD. *p<0.05 (G) TP53 expression plasmid (0.1 µg/µL) was cotransfected with pp53 TA Luc (0.25 µg/mL) plasmid into ES-2 cells mutated in *TP53*. The addition of DS-7423 increased the relative luciferase activity of TP53 in a dose-dependent manner. The experiments were repeated 3 times, and each value is shown as the mean of 3 experiments ± SD. *p<0.05.

The phosphorylation of MDM2 is associated with the activation of MDM2 and degradation of TP53, with the phosphorylation of TP53 at Ser46 playing a key event in the TP53-dependent apoptosis (28). Treatment with DS-7423 reduced the level of p-MDM2 in a dose-dependent manner (Figure 3A and 6A). Inversely, DS-7423 increased TP53 level even at lower doses, resulting in increased expression of p-TP53 (Ser15 and Ser46) (Figure 6A). However, only p-TP53 (Ser46), not p-TP53 (Ser15), was clearly induced by high doses of DS-7423 (156–2,500 nM). We then used semi-quantitative RT-PCR to evaluate the regulation of genes that are directly regulated by TP53 in OVMANA and OVISE cells. DS-7423 induced the expression of the pro-apoptotic genes *p53AIP1* and *PUMA* at 39 nM or higher doses, but did not induce the expression of p21 at any of the three doses tested (39, 156, and 2,500 nM) (Figure. 6B and 6C). We also performed semi-quantitative RT-PCR of other TP53 target genes involved in DNA repair (p53R2), metabolism (TIGAR and GLS2), G2/M arrest (GADD45), and cell cycle arrest/senescence (14-3-3 sigma and PAI-1) to test whether other TP53 target genes are induced by DS-7423. GADD45 was significantly induced by DS-7423 in OVISE cells (Figure 6B and 6C), in which G2/M arrest was enhanced by DS-7423 according to the MTT assay (Figure 4A). The other TP53-downstream genes tested were not induced by DS-7423 in both OVISE and OVMANA cells, and expression of TIGAR was rather decreased in OVMANA cells (Figure S4).

### TP53 activation is responsible for DS-7423-mediated apoptosis

We used siRNAs specific to *TP53* to knockdown *TP53* expression in OVISE cells, and treated the cells with DS-7423 at either 156 or 2,500 nM. The size of the population of apoptotic cells was calculated by annexin-V FITC–PI double staining 48 h after treatment of DS-7423. Knockdown of TP53 levels rescued cells from apoptotic cell death induced by treatment with both DS-7423 doses (Figure 6D). Immunoblotting indicated that two independent siRNAs (siRNA1 and siRNA2) specific to TP53 suppressed the expression of TP53 by >80% (Figure 6E). Next, we performed the MTT assay by applying both DS-7423 and siRNA to TP53 in OVISE cells (wild-type TP53). The anti-proliferative effect of DS-7423 was significantly reduced when combined with the knockdown of TP53 (Figure 6F). The effect of DS-7423 on the transcriptional activity of TP53 was also examined by luciferase assays in ES-2 cells with mutations in *TP53*. The cells were treated with DS-7423 for 24 h at the indicated doses, and then cotransfected with both pp53-TA-luc plasmid (containing TP53 binding sites) and a plasmid that encodes TP53. The relative luciferase activity of TP53 was significantly enhanced by DS-7423 in a dose-dependent manner (Figure 6G).

## Discussion

The effects of the PI3K/mTOR inhibitor, DS-7423, on OCCA cell lines were examined with a particular focus on (i) the anti-proliferative effect of DS-7423, (ii) the induction of apoptosis by DS-7423, and (iii) the identification of predictive biomarkers for (i) and (ii).

MTT assays revealed a clear dose-dependent effect of DS-7423 on cell proliferation, with all nine OCCA cell lines displaying sensitivity to DS-7423 (IC_50_ at 75 nM or lower), regardless of mutations on *PIK3CA*. The sensitivity to DS-7423 was significantly higher in OCCA than in OSA cell lines. The prevalence in OCCA cell lines of activating mutations in genes that encode components of the RTK-PI3K-AKT signaling pathway might account, at least in part, for their broad sensitivity to DS-7423. Differences in the dose-dependence of the anti-proliferative effects of DS-7423 and rapamycin suggest differences in the modes of action of these two drugs. Whereas DS-7423 showed a more robust anti-proliferative effect at the higher concentrations tested (>40 nM), rapamycin suppressed cell proliferation even at lower concentrations (<10 nM), and concentrations >10 nM failed to suppress the proliferation any further. This dose dependency is compatible with the phosphorylation levels of the target proteins in immunoblotting data and several previous reports in other types of cancers [10], [12], [36]. The cell cycle profile was distinct among each cell line. For example, G1 arrest was not induced and G2/M ratio was high in OVISE cells under DS-7423 exposure. This might be partly explained by the fact that GADD45 was induced by DS-7423 in these cells. Thus, the action mechanism of DS-7423 might be distinct in each type of cells, regardless of the TP53 status. Resistance to mTOR (mTORC1) inhibitors might be induced by several mechanisms, including increased activity of another mTOR complex, mTORC2, or upregulation of receptor tyrosine kinases such as insulin-like growth factor-1 receptor (IGF-R1) [37], [38]. The use of mTORC1 inhibitors to treat OCCAs is currently being investigated in phase 2 clinical trials. The currently ongoing GOG (Gynecologic Oncology Group)-0268 (NCT01196429) trial recruits OCCA patients and treats the subjects with carboplatin and paclitaxel, followed by temsirolimus (CCI-779). A report on six cases with weekly administration of temsirolimus in recurrent OCCA patients showed partial response in one patient and stable disease in another patient [39]. However, given that our data suggest that dual PI3K/mTOR inhibitors, such as DS-7423, might be more promising than single mTORC1 inhibitors, clinical trials that involve a dual PI3K/mTOR inhibitor, such as DS-7423, seem warranted for OCCA.

DS-7423 induced significantly higher levels of apoptotic cell death in OCCA cells without mutations in *TP53* than in OCCA cells with *TP53* mutations. This result suggests both that the mutational status of *TP53* might be a good biomarker to predict apoptosis induction by DS-7423, and that apoptosis depends on TP53 function. TP53 is degraded by MDM2, a ubiquitin ligase for TP53, and the MDM2 function is augmented by the kinase activity of Akt. Akt-mediated phosphorylation of MDM2 blocks its binding to p19ARF, increasing the degradation of TP53 [40], [41]. DS-7423 increased the level of p-TP53 at Ser46, which results in induction of *p53AIP1* and *PUMA* (genes involved in TP53-mediated apoptosis) [29], [42]–[44]. This data suggests that the apoptotic effect of DS-7423 depends, at least in part, on TP53 activity. The reasons for p-TP53 (Ser46), not p-TP53 (Ser15), being clearly induced and for apoptosis being preferentially induced by high doses of DS-7423 should be further clarified. In addition, other non-apoptotic genes were not significantly induced by DS-7423, except for GADD45 in OVISE cells. Further analyses are warranted whether TP53 function is more involved in apoptosis rather than in cell cycle arrest and/or DNA repair process by DS-7423. Another possibility is that other proteins (such as FOXOs) which act downstream of Akt might also play a role in the induction [45]. Dephosphorylation of FOXOs at their Akt sites induces their nuclear translocation and triggers apoptosis by induction of prosurvival genes of the BCL2 family [46], [47]. The observation that the phosphorylation of FOXO1/3a was suppressed by DS-7423, regardless of TP53 status, suggests that the pro-apoptotic effect of DS-7423 cannot be explained exclusively by the phosphorylation of FOXOs. The use of siRNA to knockdown TP53 rescued OCCA cells from apoptosis caused by DS-7423. We also confirmed by MTT assay that the anti-proliferative effect of DS-7423 was significantly diminished by knocking down TP53, suggesting that intact TP53 function might enhance the anti-tumor effect of DS-7423. Recently, it was reported that cell death caused by a PI3K inhibitor, BKM-120, was associated with TP53 status in glioma cells [48], and that PI3K/AKT inhibition was suggested to induce TP53-dependent apoptosis in HTLV-1-transformed cells [49]. These data also support the importance of wild-type TP53 in the induction of the cytotoxic effect of PI3K pathway inhibitors.

The involvement of multiple molecules in the activation of the PI3K/mTOR pathway underscores the critical need to develop predictive biomarkers that might also serve as therapeutic targets. Mutations of *PIK3CA* and amplification of *HER2* have been proposed to be useful biomarkers in breast cancer [50], [51], whereas mutant Ras has been suggested to be a biomarker of resistance in several solid tumor cells [52]. All these biomarkers (*PIK3CA*, HER2 and Ras) are focused on the RTK/Ras/PI3K pathway itself, and not on the cytotoxic effects associated with PI3K/mTOR inhibitors. Our data suggest that the presence of *PIK3CA* mutation and any other PI3K-activating alteration alone might not predict the sensitivity of OCCA cells to DS-7423. ES-2 cells, with no mutations in the RTK/Ras/PI3K pathway genes examined, showed low level of p-Akt, and the effect of DS-7423 in ES-2 xenografts was less robust, suggesting that the level of PI3K pathway activation would still be important for the sensitivity. However, the mutational status of TP53 might represent a better biomarker for the selection of tumors that could be killed by DS-7423 treatment. The frequency of mutations in *TP53* in OCCA was much less frequent than for ovarian cancers with other histology types [15], [53]. These results indicate that OCCAs would be good candidates for clinical studies on the dual PI3K/mTOR inhibitor, DS-7423.

Our study has several limitations. First, cytostatic effect is still essential to suppress cell proliferation, regardless of TP53 status. Second, the ratio of apoptotic cells is low (less than 20%) even at high concentrations of DS-7423. Third, the mechanism of cytostatic effect by DS-7423 in OCCA is cell type dependent (i.e. G1 arrest was not induced in OVISE and ES-2 cells). Thus, careful consideration is required to evaluate the TP53-dependent cytotoxic effect of DS-7423. Further studies are warranted to elucidate the mechanism of action of DS-7423, and more efficient induction of apoptosis might be needed for clinical application of this drug in OCCA.

## Supporting Information

Figure S1 **Immunoblotting of OCCA cells (ES-2 and JHOC-9), treated with DS-7423 at concentrations ranging from 0 to 2,500** **nmol/L.** As shown in Figure 3, phosphorylation of AKT and its target proteins were downregulated by DS-7423. In ES-2 cells, basal level of p-AKT at Thr 308 was very low (as shown in Fig. 1), but p-AKT at Ser473 was clearly suppressed by DS-7423.
(PPTX)

Figure S2 ***In vivo*** **effect of DS-7423 in nude mice.** (A) Western blot of total lysates from the TOV-21G and RMG-1 xenografts. total lysates were harvested 2 and 6 h after the last drug administration of DS-7423. The levels of p-Akt (Thr-308) and p-S6 (Ser-240/244) were assessed. (B) Subcutaneous xenograft tumors in athymic BALB/c mice were established after injection of ES-2 cells. Mice were treated daily at the indicated doses (1.5, 3 or 6 mg/kg/day, totally 8 times) of DS-7423 or non-treated control. Estimated tumor volumes were smaller in mice treated daily with 6 mg/kg of DS-7423, compared to the control. Western blot of total lysates from the ES-2 xenografts (treated with 6 mg/kg of DS-7423) was also shown below.
(PPTX)

Figure S3 **The size of apoptotic cell population was compared between DS-7423 and rapamycin in OVISE cells, using annexin-V FITC and PI double staining (as shown in** **Fig.** **5A–5B** **).** The percentage of apoptotic cells was significantly higher in cells treated with DS-7423, compared with those with rapamycin.
(PPTX)

Figure S4 **Semi-quantitative RT-PCR in OVMANA and OVISE cells treated with DS-7423 at the indicated doses.** Each expression level of p53R2, TIGAR, GLS2, GADD45, 14-3-3 sigma and PAI-1 was not enhanced by DS-7423. Each experiment was repeated 3 times, and each value is shown as the mean of 3 experiments ± SD.
(PPTX)

Table S1 **Phosphorylation and mutational status in 9 OCCA cell lines.** Elevated phosphorylation of cMET, HER2 and HER3, and mutations of *PIK3CA*, *PTEN*, *KRAS* and *TP53* were listed in each cell line.
(XLSX)

## References

[1] Yuan TL, Cantley LC (2008) PI3K pathway alterations in cancer: variations on a theme. Oncogene 27: 5497–510. 18794884 10.1038/onc.2008.245 PMC3398461

[2] Jia S, Liu Z, Zhang S, Liu P, Zhang L, et al (2008) Essential roles of PI(3)K-p110beta in cell growth, metabolism and tumorigenesis. Nature 454: 776–9. 18594509 10.1038/nature07091 PMC2750091

[3] Wee S, Wiederschain D, Maira SM, Loo A, Miller C, et al (2008) PTEN-deficient cancers depend on PIK3CB. Proc Natl Acad Sci USA 105: 13057–13062. 18755892 10.1073/pnas.0802655105 PMC2529105

[4] Zoncu R, Efeyan A, Sabatini DM (2011) mTOR: from growth signal integration to cancer, diabetes and ageing. Nat Rev Mol Cell Biol 12: 21–35. 21157483 10.1038/nrm3025 PMC3390257

[5] Engelman JA (2009) Targeting PI3K signalling in cancer: opportunities, challenges and limitations. Nat Rev Cancer 9: 550–562. 19629070 10.1038/nrc2664

[6] Sabatini DM (2006) mTOR and cancer: insights into a complex relationship. Nat Rev Cancer 6: 729–734. 16915295 10.1038/nrc1974

[7] Guertin DA, Sabatini DM (2007) Defining the role of mTOR in cancer. Cancer Cell 12: 9–22. 17613433 10.1016/j.ccr.2007.05.008

[8] Mayo LD, Donner DB (2001) A phosphatidylinositol 3-kinase/Akt pathway promotes translocation of Mdm2 from the cytoplasm to the nucleus. Proc Natl Acad Sci USA 98: 11598–11603. 11504915 10.1073/pnas.181181198 PMC58775

[9] Maira SM, Stauffer F, Brueggen J, Furet P, Schnell C, et al (2008) Identification and characterization of NVP-BEZ235, a new orally available dual phosphatidylinositol 3-kinase/mammalian target of rapamycin inhibitor with potent in vivo antitumor activity. Mol Cancer Ther 7: 1851–1863. 18606717 10.1158/1535-7163.MCT-08-0017

[10] Serra V, Markman B, Scaltriti M, Eichhorn PJ, Valero V, et al (2008) NVP-BEZ235, a dual PI3K/mTOR inhibitor, prevents PI3K signaling and inhibits the growth of cancer cells with activating PI3K mutations. Cancer Res 68: 8022–8030. 18829560 10.1158/0008-5472.CAN-08-1385

[11] Cao P, Maira SM, Garcia Echeverria C, Hedley DW (2009) Activity of a novel, dual PI3-kinase/mTor inhibitor NVP-BEZ235 against primary human pancreatic cancers grown as orthotopic xenografts. Br J Cancer 100: 1267–1276. 19319133 10.1038/sj.bjc.6604995 PMC2676548

[12] Shoji K, Oda K, Kashiyama T, Ikeda Y, Nakagawa S, et al (2012) Genotype-dependent efficacy of a dual PI3K/mTOR inhibitor, NVP-BEZ235, and an mTOR inhibitor, RAD001, in endometrial carcinomas. PLoS One 7: e37431. 22662154 10.1371/journal.pone.0037431 PMC3360787

[13] Anglesio MS, Carey MS, Kobel M, Mackay H, Huntsman DG (2011) Clear cell carcinoma of the ovary: A report from the first Ovarian Clear Cell Symposium, June 24th, Gynecol Oncol 2010. 121: 407–415. 10.1016/j.ygyno.2011.01.005 21276610

[14] Takano M, Tsuda H, Sugiyama T (2012) Clear cell carcinoma of the ovary: is there a role of histology-specific treatment? J Exp Clin Cancer Res 31: 53–59. 22655678 10.1186/1756-9966-31-53 PMC3405444

[15] Bell D, Berchuck A, Birrer M, Chien J, Cramer D, et al.. (2011) Integrated genomic analyses of ovarian carcinoma. Nature 474: 609–615. Cancer Genome Atlas Research Network. 10.1038/nature10166 PMC3163504 21720365

[16] Ho ES, Lai CR, Hsieh YT, Chen JT, Lin AJ, et al (2001) p53 mutation is infrequent in clear cell carcinoma of the ovary. Gynecol Oncol 80: 189–193. 11161858 10.1006/gyno.2000.6025

[17] Kuo KT, Mao TL, Jones S, Veras E, Ayhan A, et al (2009) Frequent activating mutations of PIK3CA in ovarian clear cell carcinoma. Am J Pathol 174: 1597–1601. 19349352 10.2353/ajpath.2009.081000 PMC2671248

[18] Munksgaard PS, Blaakaer J (2012) The association between endometriosis and ovarian cancer: a review of histological, genetic and molecular alterations. Gynecol Oncol 124: 164–169. 22032835 10.1016/j.ygyno.2011.10.001

[19] Fujimura M, Katsumata N, Tsuda H, Uchi N, Miyazaki S, et al (2002) HER2 is frequently over-expressed in ovarian clear cell adenocarcinoma: possible novel treatment modality using recombinant monoclonal antibody against HER2, trastuzumab. Jpn J Cancer Res 93: 1250–1257. 12460467 10.1111/j.1349-7006.2002.tb01231.x PMC5926901

[20] Yamamoto S, Tsuda H, Miyai K, Takano M, Tamai S, et al (2011) Gene amplification and protein overexpression of MET are common events in ovarian clear-cell adenocarcinoma: their roles in tumor progression and prognostication of the patient. Mod Pathol 24: 1146–1155. 21478826 10.1038/modpathol.2011.70

[21] Yamamoto S, Tsuda H, Miyai K, Takano M, Tamai S, et al (2012) Accumulative copy number increase of MET drives tumor development and histological progression in a subset of ovarian clear-cell adenocarcinomas. Mod Pathol 25: 122–130. 21983935 10.1038/modpathol.2011.143

[22] Shaw TJ, Senterman MK, Dawson K, Crane CA, Vanderhyden BC (2004) Characterization of intraperitoneal, orthotopic, and metastatic xenograft models of human ovarian cancer. Mol Ther 10: 1032–1042. 15564135 10.1016/j.ymthe.2004.08.013

[23] Bono Y, Kyo S, Takakura M, Maida Y, Mizumoto Y, et al (2012) Creation of immortalised epithelial cells from ovarian endometrioma. Br J Cancer 106: 1205–1213. 22353808 10.1038/bjc.2012.26 PMC3304406

[24] Minaguchi T, Yoshikawa H, Oda K, Ishino T, Yasugi T, et al (2001) PTEN mutation located only outside exons 5, 6, and 7 is an independent predictor of favorable survival in endometrial carcinomas. Clin Cancer Res 7: 2636–2642. 11555573

[25] Samuels Y, Wang Z, Bardelli A, Silliman N, Ptak J, et al (2004) High frequency of mutations of the PIK3CA gene in human cancers. Science 304: 554. 15016963 10.1126/science.1096502

[26] Oda K, Stokoe D, Taketani Y, McCormick F (2005) High frequency of coexistent mutations of PIK3CA and PTEN genes in endometrial carcinoma. Cancer Res 65: 10669–10673. 16322209 10.1158/0008-5472.CAN-05-2620

[27] Oda K, Okada J, Timmerman L, Rodriguez Viciana P, Stokoe D, et al (2008) PIK3CA cooperates with other phosphatidylinositol 3′-kinase pathway mutations to effect oncogenic transformation. Cancer Res 68: 8127–8136. 18829572 10.1158/0008-5472.CAN-08-0755

[28] Nakagawa S, Yoshikawa H, Jimbo H, Onda T, Yasugi T, et al (1999) Elderly Japanese women with cervical carcinoma show higher proportions of both intermediate-risk human papillomavirus types and p53 mutations. Br J Cancer 79: 1139–1144. 10098748 10.1038/sj.bjc.6690181 PMC2362249

[29] Oda K, Arakawa H, Tanaka T, Matsuda K, Tanikawa C, et al (2000) p53AIP1, a potential mediator of p53-dependent apoptosis, and its regulation by Ser-46-phosphorylated p53. Cell 102: 849–862. 11030628 10.1016/s0092-8674(00)00073-8

[30] Hermeking H, Lengauer C, Polyak K, He TC, Zhang L, et al (1997) 14-3-3 sigma is a p53-regulated inhibitor of G2/M progression. Mol Cell 1: 3–11. 9659898 10.1016/s1097-2765(00)80002-7

[31] Zhan Q, Antinore MJ, Wang XW, Carrier F, Smith ML, et al (1999) Association with Cdc2 and inhibition of Cdc2/Cyclin B1 kinase activity by the p53-regulated protein Gadd45. Oncogene 18: 2892–2900. 10362260 10.1038/sj.onc.1202667

[32] Tanaka H, Arakawa H, Yamaguchi T, Shiraishi K, Fukuda S, et al (2000) A ribonucleotide reductase gene involved in a p53-dependent cell-cycle checkpoint for DNA damage. Nature 404: 42–49. 10716435 10.1038/35003506

[33] Kortlever RM, Higgins PJ, Bernards R (2006) Plasminogen activator inhibitor-1 is a critical downstream target of p53 in the induction of replicative senescence. Nat Cell Biol 8: 877–884. 16862142 10.1038/ncb1448 PMC2954492

[34] Bensaad K, Tsuruta A, Selak MA, Vidal MN, Nakano K, et al (2006) TIGAR, a p53-inducible regulator of glycolysis and apoptosis. Cell 126: 107–120. 16839880 10.1016/j.cell.2006.05.036

[35] Suzuki S, Tanaka T, Poyurovsky MV, Nagano H, Mayama T, et al (2010) Phosphate-activated glutaminase (GLS2), a p53-inducible regulator of glutamine metabolism and reactive oxygen species. Proc Natl Acad Sci U S A 107: 7461–7466. 20351271 10.1073/pnas.1002459107 PMC2867754

[36] Cho DC, Cohen MB, Panka DJ, Collins M, Ghebremichael M, et al (2010) The efficacy of the novel dual PI3-kinase/mTOR inhibitor NVP-BEZ235 compared with rapamycin in renal cell carcinoma. Clin Cancer Res 16: 3628–3638. 20606035 10.1158/1078-0432.CCR-09-3022 PMC2905505

[37] O'Reilly KE, Rojo F, She QB, Solit D, Mills GB, et al (2006) mTOR inhibition induces upstream receptor tyrosine kinase signaling and activates Akt. Cancer Res 66: 1500–1508. 16452206 10.1158/0008-5472.CAN-05-2925 PMC3193604

[38] Wan X, Harkavy B, Shen N, Grohar P, Helman LJ (2007) Rapamycin induces feedback activation of Akt signaling through an IGF-1R-dependent mechanism. Oncogene 26: 1932–1940. 17001314 10.1038/sj.onc.1209990

[39] Takano M, Kikuchi Y, Kudoh K, Goto T, Furuya K, et al (2011) Weekly administration of temsirolimus for heavily pretreated patients with clear cell carcinoma of the ovary: a report of six cases. Int J Clin Oncol 16: 605–609. 21243393 10.1007/s10147-010-0177-z

[40] Haupt Y, Maya R, Kazaz A, Oren M (1997) Mdm2 promotes the rapid degradation of p53. Nature 387: 296–299. 9153395 10.1038/387296a0

[41] Ogawara Y, Kishishita S, Obata T, Isazawa Y, Suzuki T, et al (2002) Akt enhances Mdm2-mediated ubiquitination and degradation of p53. J Biol Chem 277: 21843–21850. 11923280 10.1074/jbc.M109745200

[42] Nakano K, Vousden KH (2001) PUMA, a novel proapoptotic gene, is induced by p53. Mol Cell 7: 683–694. 11463392 10.1016/s1097-2765(01)00214-3

[43] Matsuda K, Yoshida K, Taya Y, Nakamura K, Nakamura Y, et al (2002) p53AIP1 regulates the mitochondrial apoptotic pathway. Cancer Res 62: 2883–2889. 12019168

[44] Vousden KH, Prives C (2009) Blinded by the Light: The Growing Complexity of p53. Cell 137: 413–421. 19410540 10.1016/j.cell.2009.04.037

[45] Fu Z, Tindall DJ (2008) FOXOs, cancer and regulation of apoptosis. Oncogene 27: 2312–2319. 18391973 10.1038/onc.2008.24 PMC2819403

[46] Rahmani M, Anderson A, Habibi JR, Crabtree TR, Mayo M, et al (2009) The BH3-only protein Bim plays a critical role in leukemia cell death triggered by concomitant inhibition of the PI3K/Akt and MEK/ERK1/2 pathways. Blood 114: 4507–4516. 19773546 10.1182/blood-2008-09-177881 PMC2777129

[47] Letai A (2006) Growth factor withdrawal and apoptosis: the middle game. Mol Cell 21: 728–730. 16543140 10.1016/j.molcel.2006.03.005

[48] Koul D, Fu J, Shen R, LaFortune TA, Wang S, et al (2012) Antitumor activity of NVP-BKM120– a selective pan class I PI3 kinase inhibitor showed differential forms of cell death based on p53 status of glioma cells. Clin Cancer Res 18: 184–195. 22065080 10.1158/1078-0432.CCR-11-1558 PMC3785365

[49] Jeong SJ, Dasgupta A, Jung KJ, Um JH, Burke A, et al (2008) PI3K/AKT inhibition induces caspase-dependent apoptosis in HTLV-1-transformed cells. Virology 370: 264–272. 17931677 10.1016/j.virol.2007.09.003 PMC2189985

[50] She QB, Chandarlapaty S, Ye Q, Lobo J, Haskell KM, et al (2008) Breast tumor cells with PI3K mutation or HER2 amplification are selectively addicted to Akt signaling. PLoS One 3: e3065. 18725974 10.1371/journal.pone.0003065 PMC2516933

[51] O'Brien C, Wallin JJ, Sampath D, GuhaThakurta D, Savage H, et al (2010) Predictive biomarkers of sensitivity to the phosphatidylinositol 3′ kinase inhibitor GDC-0941 in breast cancer preclinical models. Clin Cancer Res 16: 3670–3683. 20453058 10.1158/1078-0432.CCR-09-2828

[52] Ihle NT, Lemos R Jr, Wipf P, Yacoub A, Mitchell C, et al (2009) Mutations in the phosphatidylinositol-3-kinase pathway predict for antitumor activity of the inhibitor PX-866 whereas oncogenic Ras is a dominant predictor for resistance. Cancer Res 69: 143–150. 19117997 10.1158/0008-5472.CAN-07-6656 PMC2613546

[53] Petitjean A, Achatz MI, Borresen Dale AL, Hainaut P, et al (2005) TP53 mutations in human cancers: functional selection and impact on cancer prognosis and outcomes. Oncogene 26: 2157–2165. 10.1038/sj.onc.1210302 17401424

