# Supplementary material for: Antitumor Activity and Induction of TP53-Dependent Apoptosis toward Ovarian Clear Cell Adenocarcinoma by the Dual PI3K/mTOR Inhibitor DS-7423
Source: PLoS One. 2014 Feb 4;9(2):e87220. doi: 10.1371/journal.pone.0087220 (PMC3913610; doi:10.1371/journal.pone.0087220)
Supplement: Figure S1 — Immunoblotting of OCCA cells (ES-2 and JHOC-9), treated with DS-7423 at concentrations ranging from 0 to 2,500 nmol/L. As shown in Figure 3, phosphorylation of AKT and its target proteins were downregulated by DS-7423. In ES-2 cells, basal level of p-AKT at Thr 308 was very low (as shown in Fig. 1), but p-AKT at Ser473 was clearly suppressed by DS-7423. (PPTX) [file pone.0087220.s001.pptx]

## Slide 1
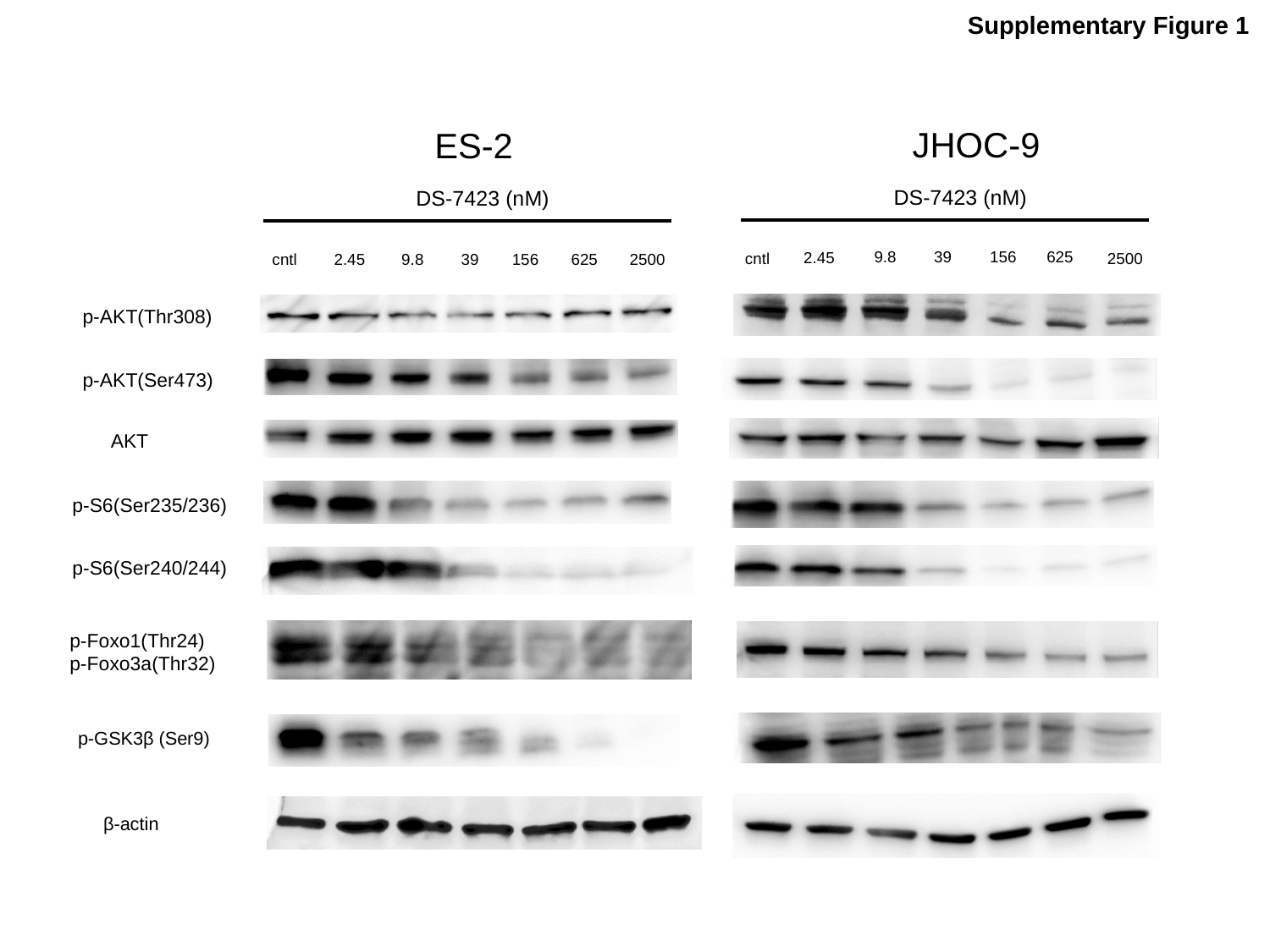

Supplementary Figure 1
JHOC-9
DS-7423 (nM)
39
156
625
9.8
2.45
cntl
2500
ES-2
DS-7423 (nM)
cntl
2.45
9.8
39
156
2500
625
p-AKT(Thr308)
p-AKT(Ser473)
AKT
p-S6(Ser235/236)
p-S6(Ser240/244)
p-Foxo1(Thr24)
p-Foxo3a(Thr32)
p-GSK3β (Ser9)
β-actin
