# Supplementary material for: Antitumor Activity and Induction of TP53-Dependent Apoptosis toward Ovarian Clear Cell Adenocarcinoma by the Dual PI3K/mTOR Inhibitor DS-7423
Source: PLoS One. 2014 Feb 4;9(2):e87220. doi: 10.1371/journal.pone.0087220 (PMC3913610; doi:10.1371/journal.pone.0087220)
Supplement: Figure S2 — In vivo effect of DS-7423 in nude mice. (A) Western blot of total lysates from the TOV-21G and RMG-1 xenografts. total lysates were harvested 2 and 6 h after the last drug administration of DS-7423. The levels of p-Akt (Thr-308) and p-S6 (Ser-240/244) were assessed. (B) Subcutaneous xenograft tumors in athymic BALB/c mice were established after injection of ES-2 cells. Mice were treated daily at the indicated doses (1.5, 3 or 6 mg/kg/day, totally 8 times) of DS-7423 or non-treated control. Estimated tumor volumes were smaller in mice treated daily with 6 mg/kg of DS-7423, compared to the control. Western blot of total lysates from the ES-2 xenografts (treated with 6 mg/kg of DS-7423) was also shown below. (PPTX) [file pone.0087220.s002.pptx]

## Slide 1
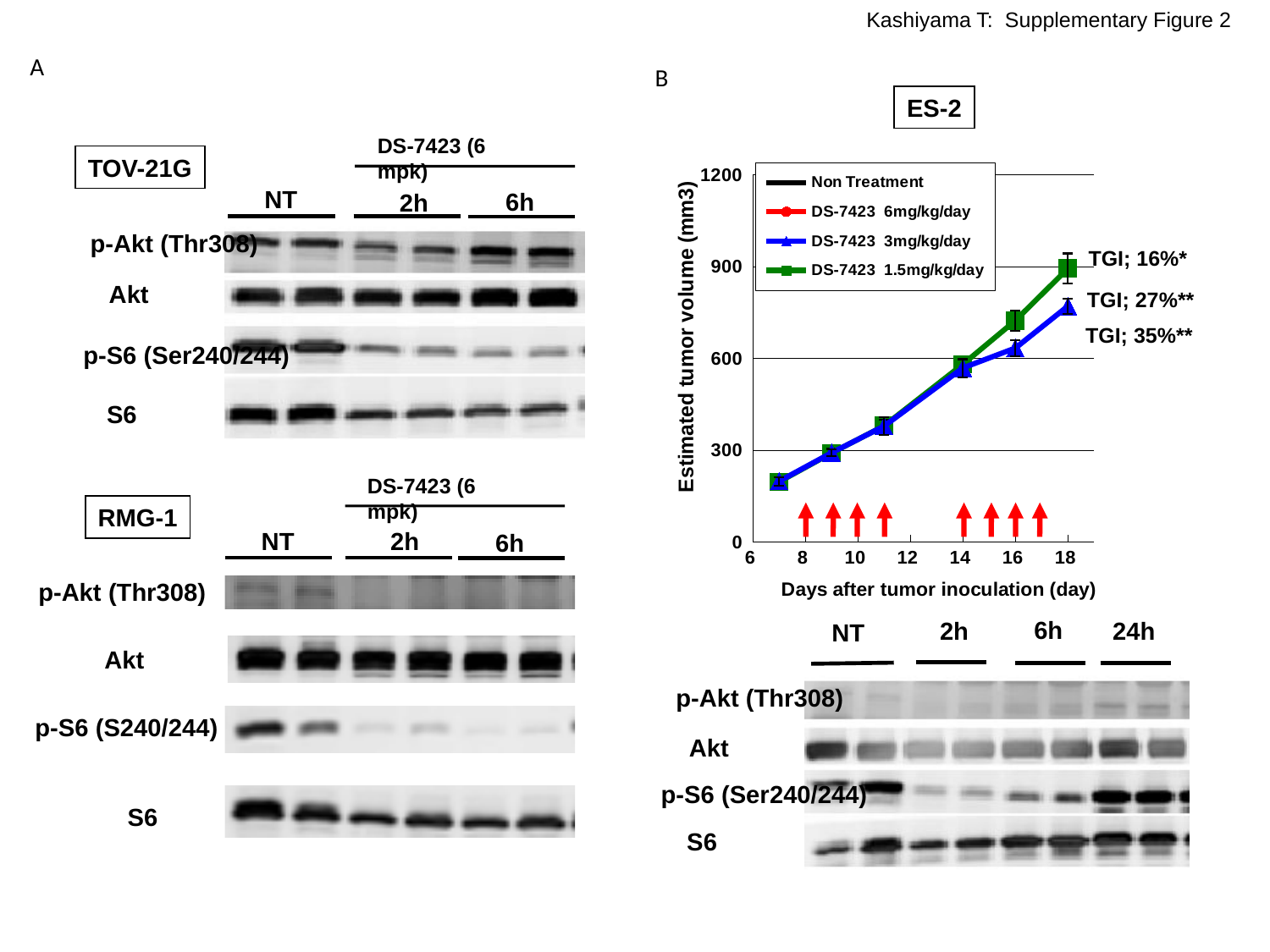

Kashiyama T: Supplementary Figure 2
A
B
ES-2
### Chart
| Category | Non Treatment | DS-7423 6mg/kg/day | DS-7423 3mg/kg/day | DS-7423 1.5mg/kg/day |
|---|---|---|---|---|DS-7423 (6 mpk)
TOV-21G
NT
6h
2h
p-Akt (Thr308)
TGI; 16%*
Akt
TGI; 27%**
TGI; 35%**
p-S6 (Ser240/244)
S6
DS-7423 (6 mpk)
RMG-1
NT
2h
6h
p-Akt (Thr308)
6h
2h
24h
NT
Akt
p-Akt (Thr308)
p-S6 (S240/244)
Akt
p-S6 (Ser240/244)
S6
S6
