# Supplementary figures and images for: Antitumor Activity and Induction of TP53-Dependent Apoptosis toward Ovarian Clear Cell Adenocarcinoma by the Dual PI3K/mTOR Inhibitor DS-7423
Source: PLoS One. 2014 Feb 4;9(2):e87220. doi: 10.1371/journal.pone.0087220 (PMC3913610; doi:10.1371/journal.pone.0087220)

## Slide 1
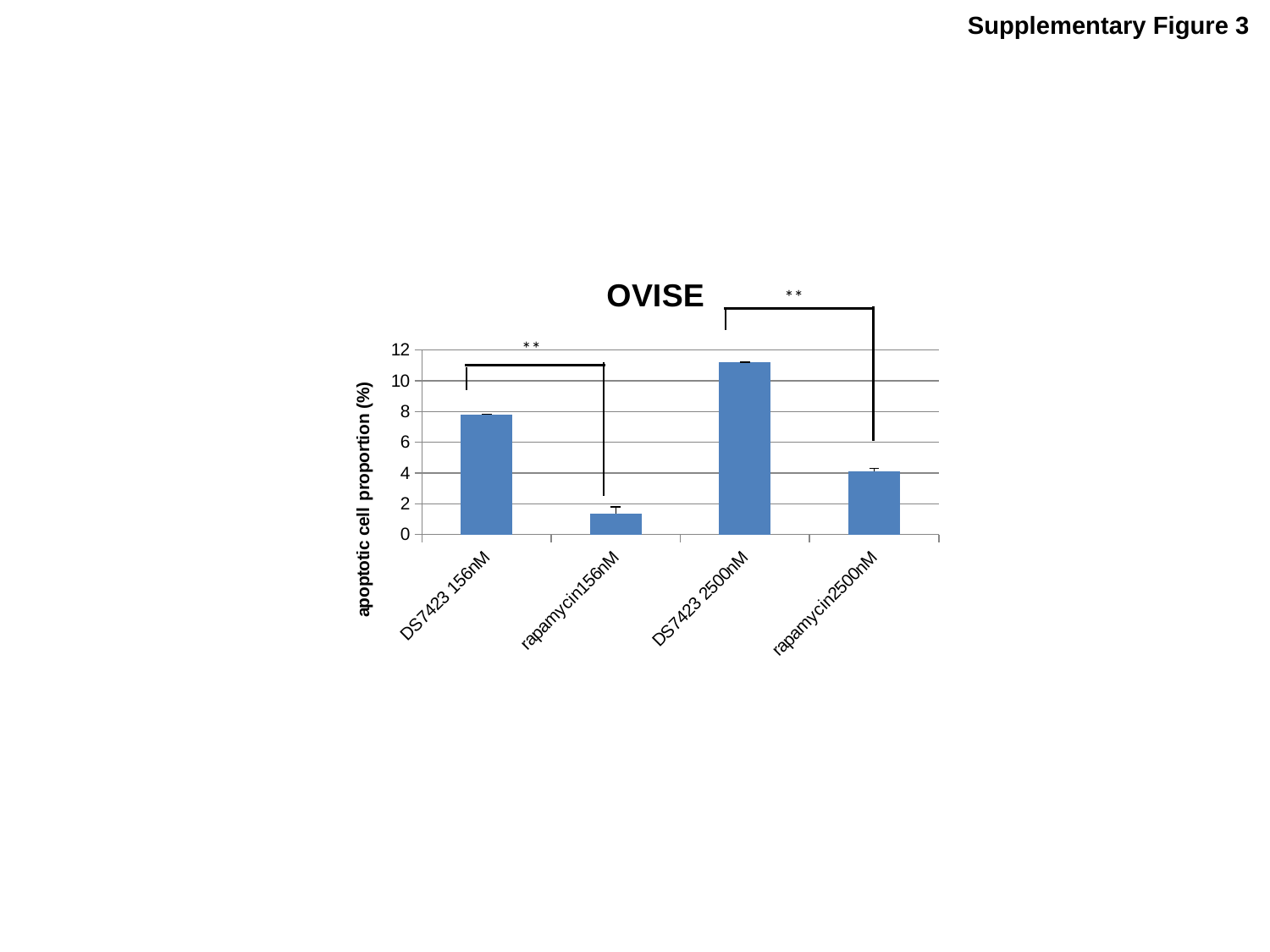

Supplementary Figure 3
### Chart: OVISE
| Category | |
|---|---|
| DS7423 156nM | 7.820000000000001 |
| rapamycin156nM | 1.3733333333333329 |
| DS7423 2500nM | 11.2 |
| rapamycin2500nM | 4.086666666666666 |**
**

Supplement: Figure S3 — The size of apoptotic cell population was compared between DS-7423 and rapamycin in OVISE cells, using annexin-V FITC and PI double staining (as shown in Fig. 5A–5B ). The percentage of apoptotic cells was significantly higher in cells treated with DS-7423, compared with those with rapamycin. (PPTX) [file pone.0087220.s003.pptx]
