# Supplementary material for: Antitumor Activity and Induction of TP53-Dependent Apoptosis toward Ovarian Clear Cell Adenocarcinoma by the Dual PI3K/mTOR Inhibitor DS-7423
Source: PLoS One. 2014 Feb 4;9(2):e87220. doi: 10.1371/journal.pone.0087220 (PMC3913610; doi:10.1371/journal.pone.0087220)
Supplement: Figure S4 — Semi-quantitative RT-PCR in OVMANA and OVISE cells treated with DS-7423 at the indicated doses. Each expression level of p53R2, TIGAR, GLS2, GADD45, 14-3-3 sigma and PAI-1 was not enhanced by DS-7423. Each experiment was repeated 3 times, and each value is shown as the mean of 3 experiments ± SD. (PPTX) [file pone.0087220.s004.pptx]

## Slide 1
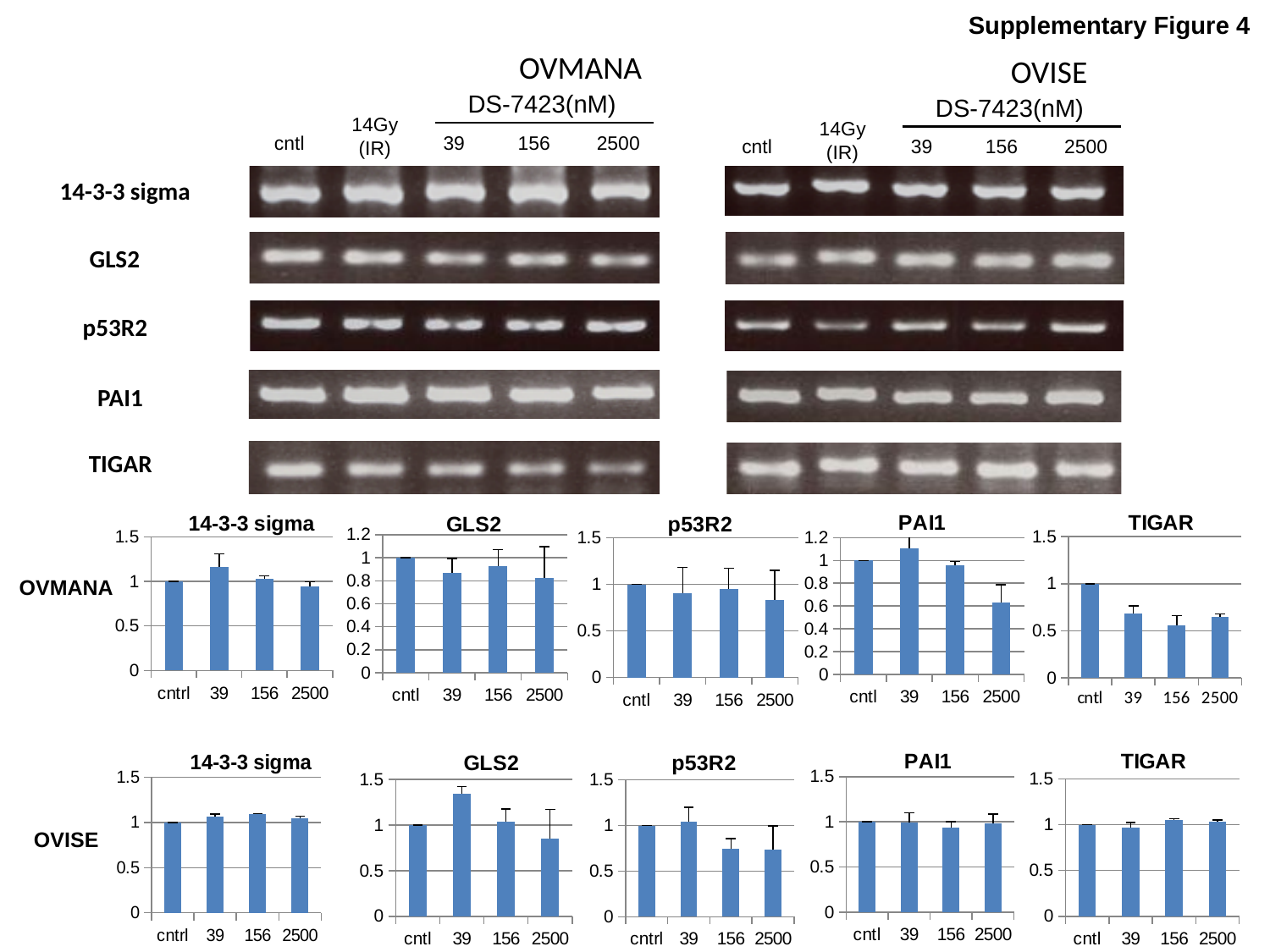

Supplementary Figure 4
OVMANA
OVISE
DS-7423(nM)
14Gy
(IR)
cntl
39
156
2500
DS-7423(nM)
14Gy
(IR)
cntl
39
156
2500
14-3-3 sigma
GLS2
p53R2
PAI1
TIGAR
### Chart: TIGAR
| Category | |
|---|---|
| cntl | 1.0 |
| 39 | 0.6834030319042729 |
| 156 | 0.5586803558382335 |
| 2500 | 0.6497381301232529 |
### Chart: PAI1
| Category | |
|---|---|
| cntl | 1.0 |
| 39 | 1.1091927802069332 |
| 156 | 0.9601234161823242 |
| 2500 | 0.6305087749178514 |
### Chart: GLS2
| Category | |
|---|---|
| cntl | 1.0 |
| 39 | 0.867216677681385 |
| 156 | 0.9272061887799953 |
| 2500 | 0.8276882367055909 |
### Chart: 14-3-3 sigma
| Category | |
|---|---|
| cntrl | 1.0 |
| 39 | 1.1652211760443139 |
| 156 | 1.03237604012553 |
| 2500 | 0.9461812011029268 |
### Chart: p53R2
| Category | |
|---|---|
| cntl | 1.0 |
| 39 | 0.9049968412548781 |
| 156 | 0.9493804115270058 |
| 2500 | 0.8340305990053304 |OVMANA
### Chart: TIGAR
| Category | |
|---|---|
| cntl | 1.0 |
| 39 | 0.9678038346200922 |
| 156 | 1.05238822571592 |
| 2500 | 1.0336842725969169 |
### Chart: p53R2
| Category | |
|---|---|
| cntrl | 1.0 |
| 39 | 1.038539432736968 |
| 156 | 0.7422784668388599 |
| 2500 | 0.7323694677148381 |
### Chart: PAI1
| Category | |
|---|---|
| cntl | 1.0 |
| 39 | 0.9929083847016259 |
| 156 | 0.9312956222584858 |
| 2500 | 0.9797525858075412 |
### Chart: GLS2
| Category | |
|---|---|
| cntl | 1.0 |
| 39 | 1.3408649662248362 |
| 156 | 1.0417901789436297 |
| 2500 | 0.8504919902220905 |
### Chart: 14-3-3 sigma
| Category | |
|---|---|
| cntrl | 1.0 |
| 39 | 1.0659151711100738 |
| 156 | 1.0904173332155402 |
| 2500 | 1.0483786242721906 |OVISE
